# Locoregional and systemic adoptive cellular therapies for pediatric brain tumors: a systematic review of CAR‑T, TCR‑engineered T cells, and NK cell strategies

**DOI:** 10.1007/s10143-026-04437-0

**Published:** 2026-08-07

**Authors:** Yaxel Levin Carrion, Jayant Bhasin, Sraavya Anne, Arman Sawhney, Caryn J. Ha, Ibraheem Sharaf, Jacob Santana, Kevin Titkov, Marvens Jean, Drew Thibault, Alejandro Pando, Nemanja Novakovic, Nehal S. Parikh, Morana Vojnic, Jonathan H. Sherman

**Affiliations:** 1 Rutgers New Jersey Medical School, Newark, NJ USA; 2 Department of Neurological Surgery, Rutgers New Jersey Medical School, 185 S Orange Ave, Newark, NJ 07103 USA; 3 Rutgers Biomedical and Health Sciences, Newark, NJ USA; 4 Department of Neurological Surgery, Corewell Health William Beaumont University Hospital, Royal Oak, MI USA; 5 Department of Pediatric Hematology and Oncology, Rutgers Robert Wood Johnson Medical School and Rutgers Cancer Institute of New Jersey, New Brunswick, NJ USA; 6 Department of Neuro Oncology, Rutgers Cancer Institute of New Jersey, New Brunswick, NJ USA; 7 Department of Neurosurgical Oncology, Rutgers Cancer Institute of New Jersey, New Brunswick, NJ USA

**Keywords:** Pediatric brain tumors, CAR-T cells, Diffuse midline glioma (DMG/DIPG), B7-H3 (CD276), GD2, Intraventricular delivery

## Abstract

Adoptive cellular therapies may expand treatment options for pediatric brain tumors by focusing activity on tumor antigens and limiting off-tumor effects. We systematically reviewed preclinical and clinical evidence for CAR T cells, TCR-engineered T cells, and NK or γδ T-cell platforms directed against HER2, B7-H3 (CD276), EGFR806-reactive EGFR, GD2, IL13Rα2, and EphA2 or EphA3, with attention to delivery route, safety, persistence, and combination strategies. Following PRISMA, we searched PubMed, Embase, and Scopus from inception through September 17, 2025, restricted to English. The search yielded 324 records; 103 duplicates were removed; 221 titles and abstracts were screened; 180 full texts were reviewed; and 34 studies were extracted by two independent reviewers. We captured design, tumor and molecular features, product engineering, route and schedule, lymphodepletion, toxicities including cytokine release syndrome, immune effector cell associated neurotoxicity, and tumor inflammation associated neurotoxicity, radiographic or clinical response, survival, and correlatives such as persistence or trafficking in blood, cerebrospinal fluid, or tumor tissue, cytokines, and antigen dynamics. In vivo studies showed reproducible antitumor activity for HER2 in medulloblastoma, GD2 in diffuse midline glioma, and multi-antigen constructs incorporating IL13Rα2 and EphA2 in medulloblastoma and ependymoma, with significant survival advantages compared with controls. γδ T cells targeting the EphA axis selectively killed medulloblastoma with neural sparing; GD2 CAR NK-92 inhibited diffuse intrinsic pontine glioma growth. In early clinical programs, route shaped safety and pharmacodynamics. For GD2, low-dose intravenous induction followed by repeated intraventricular dosing produced objective radiographic regressions and manageable tumor inflammation associated neurotoxicity, while dose-limiting cytokine release syndrome was confined to higher intravenous doses. Intraventricular B7-H3 CAR T cells, given without lymphodepletion, enabled multi-cycle dosing with mainly grade 1 to 2 events and cerebrospinal fluid localized persistence. Weekly intracranial EGFR806 CAR T cells were feasible and well tolerated, with stable disease as the best response in a small cohort. Across trials, persistence and immune activation were most evident in cerebrospinal fluid, supporting cerebrospinal fluid centered pharmacodynamic monitoring. Mechanism-based combinations, including IGF-axis inhibition in diffuse midline glioma and epigenetic priming of GD2 with an integrated safety switch in medulloblastoma, enhanced activity. The evidence supports pediatric-centric antigen selection and a CNS-first, locoregional dosing approach to increase on-tumor exposure and reduce systemic toxicity. Priorities include multi-antigen strategies to prevent escape, incorporation of safety switches, earlier deployment when tumor burden is low, and prospective cerebrospinal fluid pharmacodynamics in multisite phase II studies.

**Supplementary Information:**

The online version contains supplementary material available at 10.1007/s10143-026-04437-0.

## Introduction

Pediatric brain tumors are the most common solid malignancies in children and remain a leading cause of cancer-related mortality, with gliomas, medulloblastomas, ependymomas, and atypical teratoid or rhabdoid tumors (ATRT) representing the major histologic entities [1]. Conventional therapy is increasingly adapted and molecularly informed but still relies on a backbone of maximal safe surgical resection followed by chemotherapy, radiotherapy and in selected molecular subgroups, targeted agents. For instance, in circumscribed pediatric low-grade gliomas, maximal safe resection can provide durable disease control and may be definitive in selected completely resected tumors; however, unresectable, progressive, or infiltrative pediatric low-grade gliomas often require observation, chemotherapy, targeted therapy, or radiotherapy depending on age, location, morbidity risk, and molecular profile [2]. This distinction is important because the term “low-grade glioma” is also used for adult-type diffuse WHO grade 2 astrocytomas and oligodendrogliomas, which are not generally regarded as surgically curable despite extensive resection [3]. Targeted therapy is increasingly shaped by both mutation-defined and fusion/rearrangement-defined tumors, including BRAF V600E-mutant and BRAF-fusion or BRAF-rearranged pediatric low-grade gliomas; BRAF and MEK inhibitors show activity in selected BRAF-altered tumors, and the FDA has approved dabrafenib-trametinib for BRAF V600E-mutant disease [2, 4]. Conversely, high-grade gliomas are usually managed with surgery for diagnosis and debulking, followed by radiotherapy with or without chemotherapy in children ≥ 3 years old, whereas those < 3 years often receive chemotherapy alone to limit neurocognitive late effects. Despite these refinements, outcomes for many high risk and relapsed pediatric brain tumors remain poor, with a narrow therapeutic index for dose intensification using conventional modalities.

Against this backdrop, adoptive cellular immunotherapies have emerged as a promising strategy to exploit tumor-specific or tumor-associated antigens while potentially reducing off-target injury to the developing brain. This systematic review aims to synthesize preclinical and clinical evidence for adoptive cellular therapies in pediatric brain tumors, with emphasis on CAR‑T, TCR‑engineered T cells, and NK/γδ T cells targeting HER2, B7‑H3, EGFR806‑reactive EGFR, GD2, IL13Rα2, EphA2/EphA3, and related antigens. Here, we (*i*) compare intravenous, intraventricular, and intratumoral delivery, (*ii*) catalog acute and delayed toxicities (including cytokine release syndrome (CRS) and neuroinflammation), (*iii*) assess CAR‑cell persistence and trafficking, and (*iv*) appraise rational combinations (e.g., kinase and epigenetic inhibitors) that may widen therapeutic windows. In doing so, we anchor interpretation in both the distinctive biology of pediatric brain tumors and the rapidly expanding translational literature, from early GBM HER2‑CAR experience to ongoing pediatric trials of EGFR806, HER2, B7‑H3, and GD2 and emergent platforms such as EphA3‑CAR and γδ T cells [5–12]. Taken together, the maturing target landscape, the feasibility of repeated intracranial dosing, and the first signs of meaningful activity in diffuse midline glioma (DMG) and other pediatric CNS tumors justify a comprehensive, methodologically rigorous synthesis to guide the next generation of trials which will likely hinge on optimal antigen selection, early‑line deployment, locoregional delivery, and thoughtfully engineered combination strategies [13, 14]. 

## Methods

### Search strategy

**Fig. 1:**
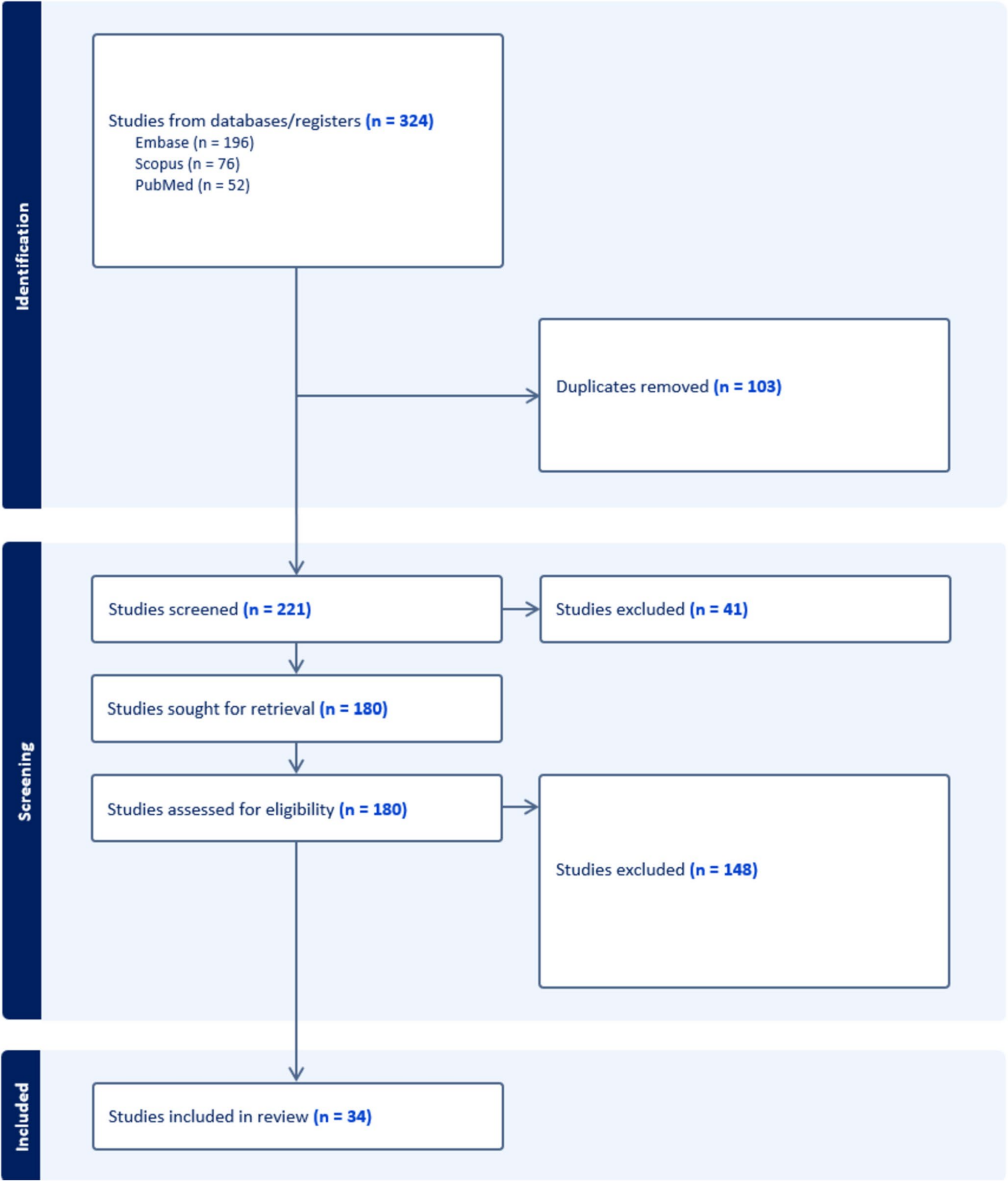
PRISMA diagram for included studies

This systematic review was conducted following PRISMA guidelines. We searched PubMed, Embase, and Scopus, which resulted in 324 total imports (52 PubMed, 196 Embase, 76 Scopus). From these, we removed 103 duplicates, and screened 221 records, of which 41 were marked as irrelevant, 180 underwent full text review, and 34 proceeded to extraction (Fig. 1). All studies were reviewed by two independent reviewers per PRISMA guidelines. The full search terms can be found in Supplemental Table 1.

### Inclusion/exclusion criteria

This review focused on research involving adoptive cellular therapies aimed at children and adolescents (age < 21 years) with primary tumors in the brain. For a study to qualify, it had to include patients with a confirmed central nervous system tumor such as diffuse intrinsic pontine glioma (DIPG), pediatric high-grade glioma, medulloblastoma, ependymoma, or ATRT. The treatment had to utilize an adoptive cell product, such as CAR-T cells, TCR-engineered T cells, or NK/γδ-based therapies (including CAR-NK), administered through any route, including intravenously, into the cerebrospinal fluid (intraventricular/intrathecal), or directly into the tumor cavity. Studies were included regardless of whether pre-infusion lymphodepletion or additional therapies were used, provided that the cellular product was the primary focus of the therapeutic intervention. Each study was required to report at least one outcome of significance, which could include safety measures (with assessments of CRS, immune effector cell‑associated neurotoxicity syndrome (ICANS), or tumor-related neuroinflammation), evidence of cell persistence in blood, CSF, or tumor tissue, tumor response and/or survival rates, or findings related to mechanistic biomarkers. We included early-phase trials, prospective and retrospective cohorts, and case series involving three or more patients. For preclinical studies, we examined in vivo models pertinent to pediatric brain tumors, such as patient-derived xenografts, syngeneic models, or genetically engineered mice, where an adoptive cell product targeting specific antigens (such as HER2, B7-H3, EGFR/EGFR806, GD2, IL13Rα2, EphA2/EphA3, or similar) was evaluated, and tumor control or survival outcomes were documented. We excluded studies that focused solely on adults, instances of metastatic brain involvement from other primary tumors, immunotherapies lacking adoptive cell components (unless paired with a qualifying cell therapy), abstracts without full text, non-English language papers, and duplicates or overlapping cohorts (retaining the most comprehensive version).

### Data extraction

For each study meeting inclusion criteria, we extracted information on study design and setting (preclinical vs. clinical, phase, single- vs. multi-center), tumor type and molecular features, patient characteristics (age, prior therapy, steroid use), and details of the cellular product (target antigen, platform [CAR-T, TCR-T- NK/γδ], costimulatory domain, backbone) along with route of administration, dosing schedule, use of lymphodepletion, and any planned combination therapies. Outcomes collected included feasibility and protocol adherence, acute and delayed toxicities (with specific capture of CRS, ICANS, and tumor-inflammation-associated neurotoxicity), radiographic and clinical responses, progression-free survival and overall survival where available, and correlative measures such as CAR-cell persistence and trafficking in blood, CSF, or tumor tissue, cytokine profiles, antigen expression or loss, and other mechanistic biomarkers in both clinical and in vivo preclinical models. Procedural considerations for repeated intracranial dosing and device management in children are detailed elsewhere [15]. 

### Data synthesis

Given the substantial clinical and methodological heterogeneity of the included studies, a quantitative meta-analysis was not performed. The evidence base comprised both preclinical in vivo studies and early-phase clinical reports, with major differences in tumor histology and molecular subtype, antigen target, cellular platform (CAR-T, TCR-engineered T cells, NK/γδ products), route and schedule of administration, use of lymphodepletion or concurrent therapies, and reported efficacy and safety outcomes. In addition, few studies evaluated the same target/platform/route/outcome combination, and effect measures were not sufficiently comparable to support valid pooled estimates. We therefore performed a structured narrative synthesis organized by target/platform, disease context, route of delivery, safety, persistence/trafficking, radiographic response, and survival.

### Risk of bias

Most included studies were preclinical experiments or small, early-phase, single-arm clinical trials from single centers, so the risk of selection and confounding bias is high. Clinical cohorts often required strict eligibility (such as antigen expression or ability to taper steroids) and many patients received radiation, chemotherapy, or targeted agents close to the time of cellular therapy, making it difficult to separate the cellular therapy from the effects of other therapies. ^6,8,16,17^ Imaging and outcome definitions were not uniform, and treatment-related inflammation (edema, tumor inflammation‑associated neurotoxicity (TIAN)) sometimes made radiographic response more difficult to interpret. We judge overall certainty as low-to-moderate for treatment- or target-specific conclusions, but stronger for qualitative patterns that appeared repeatedly, such as feasibility of repeated intracranial dosing and different toxicity profiles of intravenous vs. intraventricular administration [6, 8, 16, 17]. 

## Results

### HER2‑directed adoptive therapies (preclinical and early clinical)

Foundational preclinical work established that HER2‑reactive T cells can control medulloblastoma in vivo. In orthotopic medulloblastoma xenografts, adoptively transferred HER2‑specific T cells induced rapid tumor regression (bioluminescence decline) with a significant difference in tumor growth slopes versus controls (*P* = 0.002) and produced a survival advantage on Kaplan–Meier analysis (mean survival 115 ± 5 days in HER2‑CAR-T cell–treated mice vs. 62 ± 8 and 67 ± 10 days in controls, *P* < 0.001), corroborating true anti‑tumor activity as opposed to simply cytostatic effects [18]. Building on this, intratumoral delivery of second‑generation HER2.BBζ CAR T cells in medulloblastoma xenografts produced more pronounced tumor control than intravenous dosing(*p* < 0.0001). Tissue profiling showed brain‑localized enrichment after intratumoral dosing (trend *p* = 0.06) and low/variable detection in peripheral blood (not significant), consistent with a pharmacokinetic advantage for locoregional delivery [19]. These in vivo data align with the concept that trafficking and microenvironmental hurdles can be partly surmounted by intracranial dosing strategies. Early‑phase clinical experience with HER2‑redirected virus‑specific T cells (VSTs) in progressive glioblastoma (adolescents/young adults included) showed no dose‑limiting toxicities, radiographic regressions in a subset, and persistence by qPCR for up to 12 months in blood, which is evidence that CNS‑directed cellular products can survive and circulate after infusions [5]. We identified early‑phase pediatric programs targeting GD2, B7‑H3, EGFR806, and HER2; design features, dosing, route, and feasibility are summarized in Table 1, while tumor control and survival advantages across orthotopic models of DMG, medulloblastoma, and ependymoma are summarized in Table 2.

**Table 1:** Clinical studies & clinical operations

Study (Last name, year)	**Design & setting**	**Tumor type & molecular**	**N (enrolled/treated)**	**Age / prior therapy / steroid use**	**Cellular product (target)**	**Platform / costim / backbone**	**Route & dosing schedule**	**Lymphodepletion**
(Monje, 2024) [8]	Clinical; Phase I; single‑center	H3K27‑altered DMG/DIPG ± spinal DMG	13/ 11	2–30 years; completed frontline radiotherapy ≥4 weeks prior; not on corticosteroids at enrollment	GD2 CAR‑T	Retroviral GD2.4-1BB.CD3ζ, iCasp9 safety switch; manufactured with IL-7/IL-15 and dasatinib	IV single dose (DL1: 1×10^6/kg; DL2: 3×10^6/kg), then repeated ICV infusions (10–30×10^6 cells) every 1–3 months if benefit	None for ICV; IV per protocol
(Vitanza, 2025) [17]	Clinical; Phase I; single‑center	DIPG/DMG	23 / 21	Median age 6y (range 2–22); all post‑radiotherapy; 57% treated after progression; dexamethasone ≤2.5 mg/m²/day allowed	B7‑H3 CAR‑T	Second‑generation CAR, 4‑1BB:ζ backbone; retroviral vector with EGFRt tag; huDHFRFS for ex vivo enrichment	ICV infusions via CNS catheter; q2w × 2 courses (DLT window), then q2–4w; intra‑patient dose escalation (DR1–DR4: 1×10^7 → 10×10^7 cells per dose)	None
(Gust, 2025) [6]	Clinical; Phase I; single‑center	Pediatric CNS tumors (high‑grade glioma, ATRT, ependymoma, medulloblastoma); EGFR overexpression required by IHC (>10% cells, ≥2+ staining)	11 / 4	1–26 years; all heavily pretreated (surgery, chemo, RT); steroids ≤2.5 mg/m²/day allowed if stable ≥1 week	EGFR806 CAR‑T	Second‑generation CAR: IgG4 hinge, CD28 TM, 4‑1BB:ζ signaling; retroviral vector; truncated EGFR marker	Weekly intracranial infusions via resection cavity (Arm A) or lateral ventricle (Arm B); DR1: 4 doses at DL1 (1×10^7), then DL2 (2.5×10^7); planned escalation to DL3/DL4 not reached	None
(Vitanza, 2021) [30]	Phase I, single‑institution	Pediatric CNS tumors with HER2	3	All <19 at diagnosis; heavily pretreated (≥3 surgeries, ≥1 RT, ≥1 chemo); steroid use not detailed, but patients able to self‑report neurologic changes	HER2 CAR‑T	Second‑generation CAR, trastuzumab scFv, 4‑1BB:ζ signaling; retroviral vector; medium‑length IgG4 hinge spacer; EGFRt tag for selection	Locoregional infusion via tumor cavity (Arm A) or ventricular catheter (Arm B); weekly ×3 per 4‑week course; intra‑patient dose escalation (DL1=1×10^7; DL2=2.5×10^7 cells)	None
(Ahmed, 2017) [5]	Clinical; Phase I; single‑center	Progressive HER2‑positive glioblastoma (adults and children)	17 / 17	Mixed; Adults (n=10) median 60y (30–69); children (n=7) median 14y (10–17); all heavily pretreated (surgery, RT, temozolomide ± salvage therapies); steroid use not detailed but Karnofsky/Lansky ≥50 required	Autologous virus‑specific T cells (CMV, EBV, Adv) genetically modified to express HER2‑CAR	Second‑generation HER2‑CAR (FRP5 scFv, CD28:ζ endodomain); retroviral vector; infused as HER2‑CAR VSTs	IV infusions; 5 dose levels (1×10^6/m² → 1×10^8/m²); repeat dosing allowed up to 6 cycles at 6–12 week intervals if clinical benefit	None
(Majzner, 2022) [16]	Phase I, single‑center ; 3+3 dose‑escalation IV with optional ICV re‑infusion	H3K27M‑mutant diffuse midline gliomas (DIPG and spinal DMG)	4 patients treated at DL1 (1×10^6/kg IV); 3 DIPG, 1 spinal DMG	5–25 years; all ≥6 months post‑radiotherapy; no corticosteroids at enrollment	GD2 CAR‑T	Retroviral GD2‑CAR (14g2a scFv, 4‑1BB:ζ signaling); iCasp9 suicide switch; manufactured with IL‑7/IL‑15 + dasatinib	IV induction (DL1: 1×10^6/kg); patients with benefit eligible for repeated ICV infusions (flat dose ~30×10^6)	Cyclophosphamide + fludarabine before IV; none before ICV
(Vitanza, 2023) – Locoregional [18]	Phase I, single‑center; outpatient locoregional CAR T infusions via intracranial catheter (Ommaya or VP shunt)	Mixed pediatric CNS tumors	62 enrolled; 41 treated; 307 intracranial doses delivered	Median age 12y (range 2–25); all post‑RT; prior therapies varied; steroid use allowed if stable (≤2 anti‑epileptics, no encephalopathy)	Multiple (HER2, EGFR806, B7‑H3)	Multiple CAR‑T platforms	ICV/IT via reservoir or programmable shunt	None
(Vitanza, 2023) – Intraventricular [7]	Phase I, single‑institution; open‑label; repetitive intraventricular CAR T dosing (Arm C: DIPG)	Diffuse intrinsic pontine glioma (DIPG); majority H3K27M‑altered DMG; pontine location required, biopsy optional	5 enrolled; 4 treated	Ages 5–22; all post‑radiotherapy; some enrolled after progression; steroid use not required/excluded, but none received high‑dose steroids during dosing	Autologous B7‑H3 CAR T cells	Second‑generation CAR, 4‑1BB:ζ signaling; retroviral vector; EGFRt tag; huDHFRFS (methotrexate‑resistant DHFR) for GMP selection	Intraventricular infusions via Ommaya reservoir; every 2 weeks; fixed DL1 (1×10^7 cells); repeated dosing feasible (up to 18 infusions in one patient)	None (not feasible for repeated locoregional dosing)
Study (Last name, year)	**Planned combinations**	**Feasibility / adherence**	**Acute toxicities (CRS / ICANS / TIAN)**	**Delayed tox**	**Radiographic & clinical responses**	**PFS / OS**	**Correlative persistence / trafficking & other biomarkers**	
(Monje, 2024) [8]	None in‑arm	Serial ICV feasible; protocolized neuro‑ICU monitoring	IV: CRS in all; grade 4 CRS as DLT at DL2; ICANS at DL1 (1/3, grade 2) and DL2 (4/8; 1 grade 3); TIAN in 91% post-IV. ICV: CRS in 34% infusions (mostly grade 1–2), no ICANS; TIAN in 71% infusions, decreasing with repeated dosing	One grade 5 intratumoral hemorrhage in region of known vascular anomaly; one reversible grade 3 peripheral sensory neuropathy; no painful GD2-related neuropathy	4 major volumetric regressions (52%, 54%, 91%, 100%); 1 ongoing CR >30 mo; 9 with neurological benefit	Median OS 20.6 months from diagnosis overall; DIPG 17.6 months; sDMG 32.0 months (exploratory; no control arm)	CAR expansion in blood post-IV and detectable in CSF post-ICV; higher plasma IL-2 associated with response; CSF cytokine surges post-ICV; rising CSF TGF-β trends with progression; CSF H3K27M ctDNA decreases with response and rises with progression	
(Vitanza, 2025) [17]	None in‑arm	Repeated ICV courses feasible via reservoir	Common: headache, nausea/vomiting, fatigue, fever; mostly grade 1–2. One DLT: grade 4 intratumoral hemorrhage. No ICANS; fever considered local immune activation, not systemic CRS	One grade 3 hydrocephalus; otherwise limited delayed events	Best MRI responses: 1 partial response (6%), 15 stable disease (83%), 2 progression (11%). Clinical benefit in some patients; one long‑term decrease in pontine T2 hyperintensity	Median OS from diagnosis: 19.8 months; median survival from CAR T infusion: 10.7 months. 3 patients alive at 44–52 months; survival superior to historical median (~11 months)	CAR T detected in CSF in 72% of evaluable patients; rare in blood. CSF cytokines (CXCL10, IFNγ, GM‑CSF, TARC) elevated post‑infusion; CRP/SAA rose with repeated dosing. Correlatives support local immune activation	
(Gust, 2025) [6]	None in‑arm	All planned intracranial infusions delivered	No dose‑limiting toxicities; all ≤grade 2. Common: headache, nausea/vomiting. One grade 1 seizure. Neurologic changes (paresthesia, weakness, urinary changes) in 3 patients (grades 1–2). No CRS; systemic events mild (fatigue, fever, tachycardia). TIAN retrospectively graded: mostly grade 1–2	None >grade 2 attributable; one unrelated grade 3 lymphopenia	3/4 progressed; 1 spinal DMG patient had possible pseudoprogression (edema) → stable disease → complete response after salvage chemo	Not reached; best response stable disease; most patients relapsed/progressed; one long‑term CR after chemo	No CAR detected in CSF; CAR transcripts detected in blood in 2/4 patients (qPCR); cytokine surges (IL‑6, IL‑8, IFNγ) in CSF in some cases; blood cytokines not consistent with CRS; EGFR positivity enriched in high‑grade gliomas (41% of HGG samples)	
(Vitanza, 2021) [30]	None	Feasibility of repeated intracranial dosing established	No dose‑limiting toxicities; common: headache, pain, transient worsening of baseline deficits; fever in 2/3 (Arm B); CRP elevations correlated with cytokine surges; no systemic CRS/ICANS; local inflammatory changes on MRI	None reported beyond transient neurologic symptoms; quality‑of‑life assessments ongoing	At Course 2: 1 stable disease, 2 progressive disease; clinical symptoms correlated with CSF cytokine elevations and imaging inflammation	Not mature; interim feasibility/safety focus; survival outcomes not pooled	No CAR detected in CSF/blood; but non‑CAR T cells increased in CSF post‑infusion; CSF cytokines (CXCL10, CCL2) markedly elevated; serum CRP correlated with CSF cytokines and clinical symptoms; MRI showed peri‑tumoral edema consistent with local immune activation	
(Ahmed, 2017) [5]	None	Protocol feasible; no DLTs	No dose‑limiting toxicities; grade 2 seizures/headaches in 2 patients; otherwise well tolerated; no CRS/ICANS	None severe; hematologic toxicities (lymphopenia, neutropenia) observed but mostly unrelated; one grade 4 cerebral edema reported	Of 16 evaluable: 1 partial response (>9 months), 7 stable disease (8 weeks–29 months), 8 progressed; 3 patients alive without progression at 24–29 months	Median OS 11.1 months from infusion; 24.5 months from diagnosis; patients without prior salvage therapy had longer OS (27.2 vs 6.7 months)	HER2‑CAR VSTs detectable in blood up to 12 months (low frequency, no expansion); IFN‑γ ELISPOT confirmed virus‑specific activity; pseudoprogression suspected in some cases (peri‑tumoral edema); persistence limited without lymphodepletion or viral antigen boost	
(Majzner, 2022) [16]	None concurrent; protocol allowed re‑irradiation between cycles	Manufacturing successful for all; Ommaya reservoir required for ICP monitoring; intensive neuro‑ICU precautions	IV: CRS in all (grade 1–3); ICANS in 2 patients (grade 2–3); TIAN common (brainstem edema, hydrocephalus, cranial nerve worsening). ICV: CRS milder; TIAN frequent but manageable with ICP drainage, anakinra, steroids	One grade 5 intratumoral hemorrhage (vascular anomaly, DIPG natural history); no off‑tumor GD2 neuropathy	3/4 showed radiographic and clinical improvement; volumetric reductions up to 27%; neurological gains (facial strength, gait, motor function)	Survival varied: DIPG patients lived 13–26 months from diagnosis; spinal DMG patient ~20 months; exploratory only	CAR expansion in blood post‑IV; CAR detected in CSF post‑ICV; CSF cytokine surges (IFNγ, TNF, IL‑2, IL‑6) after ICV; CSF H3K27M ctDNA decreased with response, rose with progression; single‑cell RNAseq showed myeloid heterogeneity and Treg dynamics	
(Vitanza, 2023) – Locoregional [18]	None concurrent	Manufacturing successful; 90% infusions outpatient; up to 17 doses per patient; no catheter removals required	No catheter‑related SAEs; no meningitis, hematomas, or allergic reactions; no CRS/ICANS attributable to locoregional dosing; TIAN managed with ICP monitoring and shunt adjustments	None significant; one shunt skin breakdown (unrelated to CAR T); no reservoir removals	Early feasibility/safety focus; radiographic responses not pooled; stable disease predominated; quality‑of‑life preserved with outpatient dosing	Not reported (safety/feasibility study); survival outcomes to be reported in target‑specific manuscripts	CSF samples collected for cytokines, cytology, proteogenomics; CAR T detected in CSF; ongoing work on optimal delivery point and timing; glymphatic trafficking under investigation	
(Vitanza, 2023) – Intraventricular [7]	None concurrent; re‑irradiation allowed outside protocol	Feasibility procedures and clinical monitoring	Most common: headache, nausea/vomiting, fever; all grade 1–2, self‑limited; one transient worsening of pontine symptoms; no dose‑limiting toxicities; no CRS/ICANS requiring intervention; TIAN (tumor inflammation‑associated neurotoxicity) observed but reversible	None severe; no ICU admissions; no use of cytokine antagonists or steroids	1 patient (S008) had sustained radiographic improvement (19.4% tumor reduction, improved cranial nerve palsy) through 12 months; 2 patients (S005, S014) progressed radiographically but remained alive >12 months post‑infusion	S005 alive 17 months from enrollment; S008 alive 16 months; S014 alive 12 months; survival extended compared to historical DIPG median (~11 months)	CAR T detected in CSF (up to 64% of lymphocytes, predominantly CD8+); absent in blood; CSF cytokine surges (CCL2, CXCL10, IFNγ, GM‑CSF, TNFα) post‑infusion; targeted mass spectrometry showed modulation of B7‑H3, CD14, CD163, CSF‑1, CXCL13, VCAM‑1; serum B7‑H3 declined over time; CSF proteomics supported local immune activation	

**Table 2:** Preclinical/in-vivo studies

Study (Last name, year)	**Model & setting**	**Tumor type & molecular**	**Cellular product (target)**	**Platform / costim**	**Route & schedule**
(Ahmed, 2007) [19]	Orthotopic xenograft (mouse)	Medulloblastoma; ~40% express HER2 at low levels. HER2 expression confirmed on CD133⁺ progenitor cells.	HER2‑reactive T cells	CAR‑T; 1st‑gen	Intratumoral injection of 2×10⁶ HER2-CAR T cells into established tumors, single administration (day 5 post tumor inoculation)
(Nellan, 2018) [20]	Orthotopic xenograft	Pediatric medulloblastoma; HER2 overexpressed in ~40% of cases; HER2 surface expression linked to worse survival	HER2 CAR‑T (4‑1BBζ)	CAR‑T; 2nd‑gen	- Regional (intratumoral/intraventricular): 5×10⁵ CAR T cells - Intravenous: 2.5×10⁶ CAR T cells (higher dose required for efficacy)
(Mount, 2018) [21]	Orthotopic PDX	Pediatric diffuse midline gliomas (DMG) with H3‑K27M mutation (DIPG, thalamic, spinal cord). GD2 uniformly and highly expressed in all H3‑K27M+ cultures; low in H3‑WT pHGG	GD2 CAR‑T	CAR‑T; 2nd‑gen	Systemic IV infusion of 1×10⁷ CAR T cells (single dose) into NSG mice bearing orthotopic xenografts
(Donovan, 2020) [27]	orthotopic xenografts of group 3 medulloblastoma and PFA ependymoma in NSG mice	Pediatric group 3 medulloblastoma and posterior fossa group A (PFA) ependymoma; targets EPHA2, HER2, IL‑13Rα2 consistently expressed across primary, metastatic, recurrent tumors	IL13Rα2, EphA2, HER2 CARs (mono/tri)	CAR‑T; 2nd‑gen	- Intraventricular (CSF) delivery via lateral ventricle (single or repeat doses) - Compared with IV infusion (tail vein)
(Mehta, 2025) [33]	Orthotopic PDX (NSG‑PFASJ1)	Ependymoma (PFA)	B7‑H3 CAR‑T	CAR‑T; 2nd‑gen	Locoregional
(de Billy, 2022) [32]	Patient-derived DIPG/DMG cell lines, 3D neurospheres, whole brain organotypic slice cultures, and orthotopic xenograft mouse models	H3K27M DMG	GD2-CAR T cells (targeting disialoganglioside GD2)	GD2-CAR construct with CD28 + 4-1BB costimulatory domains, ζ signaling	Systemic administration of GD2-CAR T cells in xenograft mice; linsitinib given orally (dose escalation 12.5–50 mg/kg)
(Ciccone, 2024) [35]	MB cell lines (DAOY, D283 Med, Med-411 FH), patient-derived xenografts (PDX), and orthotopic xenograft mouse models	Medulloblastoma	GD2-directed CAR T cells (CAR.GD2)	Third-generation CAR: scFv 14.G2a anti-GD2, CD28 + 4-1BB costimulatory domains, CD3ζ signaling, plus inducible caspase-9 (iC9) suicide gene	Intravenous infusion of CAR.GD2 T cells in orthotopic MB mouse models; AP1903 (dimerizing drug) administered intraperitoneally for suicide switch activation; tazemetostat given orally (400 mg/kg, BID × 10 days) in combination experiments
(Lertsumitkul, 2024) [9]	in vitro assays with human GBM & DMG cell lines; orthotopic xenograft NSG mouse models (adult GBM and pediatric DMG)	High-grade gliomas: glioblastoma (GBM, adult) and diffuse midline glioma (DMG, H3K27M-mutant, pediatric)	EphA3 CAR‑T	CAR‑T; 2nd‑gen	Single intravenous infusion of CAR T cells (1×10^7 total, 1:1 CD4+:CD8+) in xenograft mice
(Boutin, 2025) [10]	In vitro MB cell lines (Grp3, Grp4, SHH), patient-derived xenografts (PDX), induced pluripotent stem cell–derived neurons & neuroepithelial stem cells; ex vivo expanded γδT cells from healthy donors	Medulloblastoma	γδ T cells (EphA2 / phosphoantigen‑BTN axis)	γδ adoptive	Ex vivo γδT cells co-cultured with MB cells (monolayer and spheroid assays); zoledronate pretreatment (1–50 µM) enhanced Vγ9Vδ2 activation
(Zuo, 2023) [28]	In vitro cytotoxicity assays with patient-derived DIPG cells and pontine progenitor controls; orthotopic xenograft mouse models (TT150630 high-GD2, TT190326 low-GD2)	Pediatric diffuse intrinsic pontine glioma (DIPG); H3K27M-mutant background; GD2 expression heterogeneity (high vs. low)	CAR‑NK‑92 (GD2)	CAR‑NK (NK‑92)	Intracerebroventricular injection (weekly × 2) of 3×10^6 GD2-CAR NK-92 cells in xenograft mice
Study (Last name, year)	**Planned combinations**	**Key efficacy outcomes (stats where available)**	**Toxicity**	**Persistence / trafficking**	**Biomarkers / mechanistic notes**
(Ahmed, 2007) [19]	None	- Ex vivo: HER2-CAR T cells secreted IFN-γ and IL-2, proliferated, and killed HER2⁺ Daoy/D283 cells; no killing of HER2⁻ D341 or autologous LCL. - In vivo: Tumor regression in all 12 treated mice (bioluminescence returned to baseline). - Survival: Mean survival 115 ± 5 days vs. 67 ± 10 (untreated) and 62 ± 8 (nontransduced T cells); P < 0.001.	No overt toxicity reported in SCID mice; no neurologic deficits or stress beyond tumor burden.	Tumors regressed but recurred in all animals → attributed to limited persistence of human T cells in SCID mice. Authors suggest enhanced CAR designs (CD28/CD134/CD137) for persistence	- HER2 expression confirmed by immunohistochemistry in 5/10 primary tumors that induced IFN-γ secretion. - HER2 expressed on CD133⁺ progenitor cells. - IL-2 production linked to NKG2D ligand expression on Daoy cells. - No costimulatory molecules (CD80, CD83, CD86, CD134, CD137) detected on tumor cells.
(Nellan, 2018) [20]	None	- In vitro: Specific killing of HER2⁺ Daoy/D283, cytokine release (IFNγ, IL‑2, TNFα) - In vivo (mouse): Complete regression of Daoy and D283 xenografts with IT delivery; IV required 5‑fold higher dose - Established tumors: Regression even in large tumors (P < 0.001 survival benefit)	NR	CAR T cells persisted at low levels in brain and blood of mice (IV and IT)	- HER2 surface expression confirmed in Daoy/D283, absent in D425 - 4‑1BB costim chosen for persistence and reduced exhaustion
(Mount, 2018) [21]	None	- In vitro: potent GD2‑dependent killing and cytokine release (IFN‑γ, IL‑2) - In vivo (DIPG xenografts): complete tumor clearance in SU‑DIPG6 and SU‑DIPG13 models; residual ~30–36 H3‑K27M⁺ GD2⁻ cells vs ~18,000–32,000 in controls - Survival (SU‑DIPG13P)*: robust improvement, P < 0.0001 vs CD19‑CAR controls	Favorable	GD2‑CAR T cells infiltrated leptomeninges and parenchyma by 7–14 days post‑treatment; remained detectable at 21 days. CD19‑CAR controls did not persist	Residual tumor cells after clearance were GD2⁻, suggesting antigen escape
(Donovan, 2020) [27]	Combination with azacytidine (DNA hypomethylating agent) to prevent antigenic escape and enhance CAR efficacy	- Group 3 MB xenografts: EPHA2 CAR significantly improved survival vs controls (P < 0.005); TRI CAR also effective but less consistent - Repeat dosing: second round of EPHA2 CAR further prolonged survival (P < 0.01) - PFA ependymoma xenografts: HER2 CAR and TRI CAR both improved survival (P < 0.05–0.005) - IV vs CSF delivery: CSF route superior (P < 0.005 across models) - Azacytidine + CAR: synergistic survival benefit, complete tumor clearance in subsets; progression‑free survival extended to >200 days in mice	No limiting toxicity reported in mice; CSF delivery well tolerated; IV less effective and associated with residual tumor burden	- CAR T cells persisted in CSF and tumor sites with repeat dosing - No antigen escape observed for EPHA2 after therapy - Azacytidine increased EPHA2 antigen expression, enhancing persistence and efficacy	- EPHA2, HER2, IL‑13Rα2 consistently expressed across primary, metastatic, recurrent MB and ependymoma - Azacytidine upregulated target antigen expression and sensitized tumors to CAR killing - Cytokine release (IL‑23, GM‑CSF) indicated enhanced T cell activation with CSF delivery - Composite CAR mixes inferior to monospecific EPHA2 CARs
(Mehta, 2025) [33]	None	Large survival benefit (log‑rank P < 0.0001); baseline tumor burden inversely correlates with benefit (r² = 0.6744; P ≤ 0.0001)	NR	NR	Early intervention may enhance benefit
(de Billy, 2022) [32]	Dual IGF1R/IR inhibitors (BMS-754807, linsitinib) with GD2-CAR T cells	- Linsitinib + GD2-CAR T cells significantly enhanced tumor cell death in vitro, ex vivo, and in vivo models - In vivo: sustained tumor control with 5×10^6 CAR T cells + linsitinib, whereas CAR T alone showed transient effect - Statistical significance: ****P < .0001 in multiple assays (cell death, confluency, infiltration)	- GD2-CAR T cells showed limited systemic/neurotoxicity in preclinical models - BMS-754807 had off-target toxicity at higher doses; linsitinib better tolerated - No major toxicity reported in xenograft mice at tested doses	- Linsitinib decreased CAR T-cell exhaustion (↓PD-1, Lag3) - Increased central memory phenotype - Enhanced infiltration of CAR T cells into DIPG neurospheres and brain slice models	- GD2 expression: heterogeneous in patient tissues, homogeneous in H3K27M-mutant cell lines - IGF1R/IR expressed and phosphorylated in DIPG cells, absent in CAR T cells - Linsitinib modulated CAR T phenotype (↓activation/exhaustion markers, ↑central memory) - Mechanism: IGF1R/IR inhibition → tumor cell death + improved CAR T persistence/infiltration
(Ciccone, 2024) [35]	EZH2 inhibitor tazemetostat to upregulate GD2 expression and sensitize GD2^dim MB cells; AP1903 for safety switch activation	- GD2 expressed in 82.7% of MB tumors (highest in SHH & G3/G4, lowest in WNT) - CAR.GD2 T cells killed GD2^+ MB cells in vitro (D283 Med: residual tumor 1.0% vs. 88.2% with NT-T, P < 0.0001) - In vivo: CAR.GD2 T cells significantly controlled tumor growth and prolonged survival in orthotopic MB models (DFS and OS curves, log-rank P ≤ 0.001) - Tazemetostat pretreatment increased GD2 MFI and improved CAR T cytotoxicity (DAOY cells: GD2^+ 33% → 79%, MFI ↑ from 1976 → 6942; enhanced killing, P ≤ 0.0001)	- No major neurotoxicity observed in MB models - GVHD-like toxicity in some mice (alopecia, weight loss) linked to donor-specific CAR T expansion - AP1903 successfully eliminated circulating and tumor-infiltrating CAR T cells, mitigating toxicity	- CAR.GD2 T cells expanded in vivo and persisted up to 45 days - Infiltrated MB tissue in xenograft models - AP1903 eliminated CAR T cells from blood and tumor, confirming BBB penetration	- GD2 heterogeneity across MB subgroups; SHH & G3/G4 most promising targets - EZH2 inhibition (tazemetostat) restored GD2 expression via GD3 synthase upregulation - Cytokine release (granzyme B, IFNγ, IL-2, TNFα) correlated with cytotoxicity - NFL (neurofilament light chain) used as biomarker of neurotoxicity; no increase in CAR.GD2-treated mice
(Lertsumitkul, 2024) [9]	None	- In vitro: robust antigen-specific cytotoxicity against GBM (U251CL) and DMG (SU-DIPG36GL) cell lines, comparable to HER2-CAR benchmark - In vivo (DMG model): complete tumor clearance in 5/7 mice (71.4%) by day 20; sustained response to day 27 - In vivo (GBM model): eradication within 4 weeks, prolonged complete response, significantly improved survival (Kaplan–Meier, P < 0.005) - Rechallenge: 100% tumor clearance in 5/5 mice on contralateral implantation; 4/5 remained tumor-free >200 days	No off-tumor toxicity observed; EphA3 absent in normal brain and vital organs (heart, liver, kidney, lung). Prior antibody studies showed favorable safety profile	- CAR T cells persisted >8 weeks in circulation - Effector memory phenotype (CD45RA–CCR7–) - Recall response on rechallenge, with rapid tumor clearance	- EphA3 highly expressed in GBM & DMG, absent in normal brain - Expression also noted in perivascular niches and glioma stem cells - Flow cytometry confirmed antigen-specific activation (↑CD137, ↑IFNγ, ↑TNFα) - Memory CAR T cells demonstrated long-term persistence and recall function
(Boutin, 2025) [10]	Amino-bisphosphonates (zoledronate) to increase phosphoantigen levels and sensitize MB cells; potential integration with adoptive γδT cell transfer	- EphA2-positive MB cells activated Vγ9Vδ1 T cells (↑CD69, P < 0.0001) - Zoledronate-treated MB cells triggered Vγ9Vδ2 degranulation and cytotoxicity (LDH release assays: SHH MB ~80% lysis vs. Grp3/Grp4 ~45%) - No cytotoxicity against healthy neurons or neuroepithelial stem cells, even at high zoledronate doses (50 µM) - Donor-dependent variability in γδT cell activation	No off-target killing of healthy neurons or stem cells; zoledronate treatment did not induce γδT-mediated cytotoxicity in non-tumor cells	γδT cells showed selective infiltration and activation in MB models; resident memory phenotype observed in endogenous MB-infiltrated γδT cells (ILR7, CCL5, GZMK expression)	- EphA2 upregulated in Grp3 and WNT MB vs. normal cerebellum - BTN3A1/BTN2A1 complex mediates Vγ9Vδ2 activation via phosphoantigen accumulation - NKG2D ligands expressed in SHH MB, but blocking NKG2D did not impair γδT cytotoxicity - Resident memory γδT phenotype in MB (PD-1^low, TIM-3^low, Ki67^low) - Selectivity confirmed: tumor-specific activation, sparing normal neural cells
(Zuo, 2023) [28]	None tested; authors note potential for future combinatorial strategies and improved animal models	- In vitro: GD2-CAR NK-92 killed high-GD2 DIPG cells (TT150630, TT150714, TT150728, DIPG17), limited activity against low-GD2 (TT190326) - In vivo: TT150630 xenografts showed significant tumor inhibition and prolonged survival (P < 0.0001 vs. controls) - TT190326 xenografts: modest tumor inhibition, no significant survival benefit	- No cytotoxicity against pontine progenitor cells - No cytokine release syndrome (CRS): plasma cytokines unchanged (IL-6, IFNγ, TNFα) - No acute injury in brain, heart, liver, spleen, lung, kidney (H&E)	- Irradiated CAR NK-92 cells lost viability rapidly (80% decline by Day 2, no activity by Day 4) - Non-irradiated CAR NK-92 persisted longer (up to Day 4 in artificial CSF), but activity declined over time	- GD2 heterogeneity critical: efficacy only in GD2-high DIPG - CAR NK-92 produced ↑IFNγ and ↑CD107a vs. unmodified NK-92 - Safety advantage over CAR-T: no CRS, no cerebral edema observed

### GD2 CAR T cells for H3K27M‑mutant diffuse midline gliomas (DMG/DIPG)

Preclinically, GD2 emerged as a uniform, high‑density target in DMG, enabling systemic CAR T‑cell eradication of established pontine and thalamic patient‑derived tumors without on‑target peripheral neuropathy; these experiments demonstrated dramatic tumor clearance and prolonged survival in multiple models [20]. This led to a first‑in‑human phase I experience at DL1 (1 × 10^6 CAR T cells/kg IV) which demonstrated TIAN managed with neurocritical care and early radiographic/clinical improvements in 3/4 patients; the program then incorporated intracerebroventricular (ICV) dosing to capitalize on CNS exposure while mitigating systemic inflammation [21]. In the completed Arm A report, which was IV induction followed by repeated ICV boosts, 13 patients were enrolled and 11 were treated (DIPG and spinal DMG) [8]. DL1 was the maximally tolerated IV dose, 3 of 8 patients at DL2 (3 × 10^6/kg IV) had dose‑limiting grade‑4 CRS, whereas no DLTs occurred with subsequent ICV infusions; importantly, all patients exhibited tumor inflammation-associated neurotoxicity (TIAN) that proved manageable with protocolized monitoring [8]. Four patients had major volumetric regressions (52%, 54%, 91%, and 100%), a complete response persisted > 30 months, and nine experienced measurable neurological benefit; correlative analyses showed blood CAR‑qPCR levels waned in tandem with rising H3K27M ctDNA in CSF at progression, supporting biological on‑treatment response tracking [8]. Notably, the pattern of toxicity differed by route: CRS was dose‑limiting after IV but infrequent after ICV, whereas TIAN accompanied both routes but diminished with repeated intracranial cycles [8]. 

### B7‑H3 CAR T cells for DIPG (locoregional only)

In a dedicated, ICV‑only phase I trial of B7‑H3 CAR T cells for DIPG, 23 enrolled/21 treated children and young adults demonstrated feasibility with repeated intracranial courses, no lymphodepletion, and an AE profile dominated by grade 1–2 events during the DLT window [16]. Median survival from first CAR T infusion was 10.7 months (range 0.6–45.8), and survival from diagnosis 19.8 months with 3 long‑term survivors, which are durations atypical for historical DIPG outcomes [16]. On imaging, the best responses among 18 evaluable patients were 1 partial response (6%), 15 stable disease (83%), and 2 progressive disease (11%); among those already progressed at entry, 88.9% attained PR/SD after initiating locoregional therapy [16]. CAR T cells were detected in 38.1% of CSF biospecimens in cycles 1–2 and in 72% of evaluable patients at ≥ 1 time point, with blood detection rare (2/96 samples; peaks ~ 205–264 copies/µg DNA by qPCR), indicating regional persistence with minimal systemic spillover [16]. The investigators contrasted their findings to the GD2 sequential IV → ICV strategy (hypothesis generating): CRS constrained IV GD2 dosing, but CRS did not complicate ICV B7‑H3 infusions; despite design and population differences, survival for treated DIPG in both programs was in a similar range (17.6 months GD2 vs. 19.8 months B7‑H3), emphasizing that route and platform choices may shape tolerability while maintaining biological activity [16]. B7-H3 shows broad, though heterogeneous, expression across pediatric CNS tumors, supporting biomarker-guided selection [22, 23]. 

### EGFR806 CAR T cells (pediatric high‑grade glioma, AT/RT and others; Phase I)

The BrainChild‑02 study tested weekly intracranial EGFR806‑CAR T infusions via resection cavity (arm A) or ventricle (arm B) without lymphodepletion in children and young adults with EGFR⁺ CNS tumors [6]. Of 11 enrolled, 4 were treated (3 HGG, 1 AT/RT) with 5–10 intracranial doses each; no dose‑limiting toxicities occurred, and the maximal AE grade was 2 (headache, nausea; isolated grade‑1 seizure; several low‑grade new neurologic symptoms). CAR transcripts became detectable in blood in 2/4 patients, indicating some systemic trafficking after intracranial dosing. The best radiographic outcome was stable disease in 1 patient (with later complete response to chemotherapy after possible immune‑related pseudoprogression), while 3 progressed on protocol [6]. Tumor tissue screening (*n* = 119 across types) corroborated EGFR immunoreactivity in substantial fractions, particularly high‑grade glioma and ependymoma, supporting target prevalence for future iterations [6]. 

### IL13Rα2‑ and EphA‑targeted platforms

Multi‑omics and field overviews highlight these antigens across diffuse midline glioma and embryonal tumors [13, 14, 24–26]. In orthotopic medulloblastoma and ependymoma models, CAR constructs directed to IL13Rα2, in multi‑antigen formats with EphA2 and HER2, consistently reduced tumor burden and extended survival versus controls, with multiple head‑to‑head comparisons reaching statistical significance (many P values < 0.05 and several < 0.01 to < 0.0001).^27^ Within the Eph family, EphA3‑CAR T cells show antigen‑dependent activity in glioma/DMG models, further supporting Eph‑directed strategies [9]. A complementary approach uses γδ T cells engaging EphA2/phosphoantigen–butyrophilin pathways to kill medulloblastoma while sparing neurons and neural stem cells, suggesting a favorable neural safety window [10]. Complementing αβ CAR‑T and γδ approaches, off‑the‑shelf CAR‑NK‑92 targeting GD2 suppressed DIPG growth in vivo;^28^ broader innate‑cell strategies for CNS delivery are reviewed elsewhere [27]. Taken together, intracranial, locoregional delivery provides a practical route to maximize on‑tumor exposure and therapeutic index for these targets [17, 28, 29]. 

### Combination strategies to overcome immune suppression

A major barrier to efficacy is IGF‑1R/insulin receptor signaling in DMG, which fuels both tumor growth and an immunosuppressive stroma. In DMG models, dual IGF‑1R/IR inhibitors (linsitinib, BMS‑754807) enhanced GD2‑CAR T cytotoxicity in vitro, ex vivo, and in orthotopic xenografts, yielding sustained antitumor effects without reducing GD2 antigen density; bar‑graph statistics met the authors’ significance thresholds across key assays, supporting a rational doublet approach [30]. 

### Toxicity, persistence, and route‑of‑administration signals across platforms

Across trials, route emerged as a dominant determinant of toxicity. In the GD2 program, CRS escalated with IV dosing, establishing DL1 (1 × 10^6/kg) as the maximally tolerated IV dose, whereas no DLTs were observed during repeated ICV boosts. In contrast, TIAN was nearly universal yet manageable, and its severity decreased with successive ICV infusions, implicating tumor debulking and/or adaptive microenvironment change [8]. EGFR806 ICV infusions were also feasible and associated with no toxicities above grade 2 in a small cohort,^6^ and B7‑H3 ICV dosing proceeded through multiple cycles without lymphodepletion, with CSF‑restricted CAR detection in most evaluable patients and minimal peripheral qPCR positivity (rare, low‑copy events), illustrating how intracranial delivery concentrates exposure where needed while lowering systemic cytokine-mediated risks [6, 16]. Taken together, these data support a sequential IV-to- ICV (for trafficking “spark” plus local consolidation) or ICV‑only paradigm depending on antigen/construct, with persistence readouts by CAR‑qPCR and CSF cytokines serving as sensitive pharmacodynamic markers [8]. 

### Early signals in embryonal histologies and ependymoma

Beyond DMG, GD2 expression and B7‑H3 ubiquity extend to medulloblastoma and ependymoma, where preclinical CAR‑mediated cytotoxicity has been reproduced. In ependymoma xenografts, B7‑H3 CAR T therapy produced a large survival benefit in an NSG‑PFASJ1 model (*P* < 0.0001) and revealed that baseline tumor burden correlated inversely with benefit (r² = 0.6744; *P* ≤ 0.0001), a practical insight for patient selection/enrichment in early trials [31]. For medulloblastoma, orthotopic models responded to HER2 CAR (Nellan, 2018; *P* < 0.0001 across tumor‑growth slopes) and to EphA2/IL13Rα2‑targeted CARs with significant event‑free survival advantages (P values < 0.0001 to 0.01 depending on construct/timing), highlighting multiple actionable antigens in embryonal tumors [19, 32]. 

## Discussion

Adoptive cellular therapies are beginning to carve out a distinct role in the management of pediatric brain tumors, but the path forward will require careful matching of platforms (CAR‑T, TCR‑engineered T cells, γδ T cells, and NK cells) to disease biology, antigens, and routes of delivery unique to children. Two themes emerge from the present corpus: first, target selection must be grounded in pediatric tumor surfaceomics and immunopeptidomics rather than extrapolated from adult glioma; second, locoregional delivery appears to be a safe and enabling technology that can convert otherwise marginal biological activity into clinically relevant signals while limiting systemic toxicities [6, 13, 17]. 

### Antigen selection in pediatric tumors

Recent multi‑omics work in DIPG/DMG gives a clear, pediatric‑focused target map. B7‑H3 (CD276), IL13Rα2, HER2, and EphA2/EphA3 appear repeatedly, and HLA peptidomics reveals cancer‑testis and endogenous retroviral peptides that could be addressed with TCR‑based approaches [24]. The current literature provides converging lines of evidence highlighting the idea that pediatric high‑grade gliomas are not simply smaller versions of adult GBM, and as such, target selection must be built around pediatric biology [14, 25]. For hemispheric H3.3‑G34–mutant gliomas, a predominantly adolescent entity, the emerging translational picture is that oncohistone‑driven epigenetics shift inflammatory programs and may render these tumors more permissive to immune attack than H3K27‑altered midline tumors, potentially broadening the window for effective cellular therapies in this genotype [33]. 

Within medulloblastoma, HER2 and GD2 are leading candidates. HER2 is expressed in a substantial subset of tumors and is absent in normal brain parenchyma, supporting a favorable on‑target/off‑tumor profile. Early preclinical studies demonstrated that HER2‑redirected T cells can eradicate orthotopic medulloblastoma and sustain responses, establishing proof‑of‑principle for therapeutic advantage [18, 19]. Contemporary work reveals that GD2 is frequently expressed across medulloblastoma subgroups and that epigenetic priming with EZH2 inhibition can upregulate GD2 on low‑expressing tumors, sensitizing them to GD2‑CAR killing. Importantly, an inducible caspase‑9 (iC9) safety switch can be pharmacologically activated across the blood–brain barrier (BBB), providing an emergency off‑switch in the central nervous system [34]. Complementing αβ CAR‑T approaches, γδ T cells recognize stress ligands such as EphA2 and phosphoantigen/Butyrophilin complexes. Recent medulloblastoma data demonstrate robust, antigen‑specific γδ cytotoxicity with sparing of neurons and neural stem cells, which is an especially attractive feature for the developing brain [10]. 

For DMG/DIPG, GD2 and B7‑H3 now have the most compelling translational arcs. A phase I program of GD2‑CAR T cells combining intravenous induction with subsequent intracranial maintenance showed symptom improvement or radiographic responses in most treated children and young adults; at the recommended intravenous dose (≈ 1 × 10^6 cells/kg), high‑grade cytokine release syndrome (CRS) was minimized, and durable complete response has been observed in at least one case [8]. In parallel, first‑in‑human intraventricular B7‑H3‑CAR dosing in patients with DIPG demonstrated feasibility, cytokine/immune activation in cerebrospinal fluid (CSF), persistent CAR detection in CSF, and durable clinical stabilization in one of the initial evaluable patients, with no dose‑limiting toxicities [7]. Ependymoma, a pediatric tumor historically resistant to systemic therapies, also appears to express B7‑H3 widely; preclinical B7‑H3‑CAR studies show potent activity, albeit with response durability inversely related to tumor burden; supporting earlier intervention or debulking to maximize CAR efficacy [31]. 

EGFR remains a rational target in pediatric high‑grade gliomas and embryonal tumors, but clinical targeting must navigate the risk of on‑target recognition of physiologic EGFR. The mAb806 epitope, exploited in EGFR806‑CAR constructs, binds a cryptic conformation preferentially exposed on tumor EGFR. In the completed BrainChild-02 pediatric phase I study using weekly locoregional infusions without lymphodepletion, EGFR806‑CAR infusions were feasible and well tolerated, with the best response of stable disease [6]. This highlights the principle that platform safety can be engineered at the binder level and that feasibility data accrued in non-pontine settings can de‑risk subsequent DMG trials. Meanwhile, new targets are entering the field: EphA3 has emerged as a consistently expressed glioma antigen absent in normal brain, and EphA3‑CAR T cells have demonstrated curative activity with memory responses in orthotopic GBM/DMG models, suggesting a strong rationale for pediatric translation [9]. 

### Delivery route and the case for locoregional dosing

While early systemic experiences, such as autologous virus‑specific, HER2‑CAR–modified T cells given intravenously without lymphodepletion, established safety and suggested benefit with peripheral persistence up to a year,^5^ several pediatric programs now favor repeated intracranial delivery to enhance on‑tumor exposure while preserving a wide therapeutic index [7, 17]. The BrainChild platform has operationalized this at scale: across three trials (HER2, EGFR806, B7‑H3), 307 intracranial CAR‑T doses were delivered to 41 children and young adults with meticulous neurosurgical workflows (Ommaya reservoirs or programmable shunts with “virtual off” settings) and a low rate of catheter‑related complications [17]. For medulloblastoma, preclinical work demonstrates that intraventricular dosing achieves regressions at log‑fold lower cell doses than intravenous infusion and was tolerable in nonhuman primates, foreshadowing the clinical feasibility now realized in pediatric trials [19]. Together, these observations suggest that locoregional dosing is not merely an alternative administration route but a core design element that can raise the ceiling on clinical effect while reducing systemic CRS and ICANS risks [6, 8, 17]. 

Importantly, successful delivery to the ventricle, CSF, or resection cavity should not be equated with uniform infiltration throughout tumor parenchyma. Clinical evidence for deep adaptive cellular infiltration into pediatric CNS tumors remains limited because most early trials rely on CSF cytokines, CSF or blood CAR-qPCR, and imaging rather than serial tumor tissue [6–8, 17, 21, 32]. The strongest proof-of-mechanism to date comes from GD2-CAR T-cell therapy in H3K27M-altered DMG, where post-mortem tissue demonstrated lymphocytic infiltration and GD2-CAR transcripts within tumor tissue but not uninvolved brain, supporting true tumor entry [14, 21]. However, even these data do not fully define penetration gradients relative to blood vessels, ependymal surfaces, infiltrative tumor margins, or catheter/cavity-adjacent regions [21]. Therefore, CSF pharmacodynamic activation should be interpreted as evidence of regional exposure and immune activation, not necessarily proof of homogeneous deep tumor infiltration [7, 8, 16, 17, 32]. Future trials should incorporate tissue-based correlatives when ethically available, including spatial immunohistochemistry, RNAscope or PCR for engineered-cell transcripts, single-cell or spatial transcriptomics, and route-specific imaging/CSF correlates to determine whether adoptively transferred cells reach deep parenchymal disease, infiltrative margins, and leptomeningeal deposits.

The specific locoregional route may also influence the pattern of tumor exposure and infiltration. Intraventricular or intrathecal dosing provides broad CSF compartment access, facilitates repeated CSF pharmacodynamic sampling, and may be best suited for diffuse midline, multifocal, ventricular, leptomeningeal, or metastatic disease [7, 8, 16, 17, 32]. However, regional CSF exposure does not necessarily prove uniform penetration into solid tumor parenchyma, because transferred cells must traffic across ependymal or pial interfaces and through the tumor microenvironment [7, 8, 17, 32]. Intracavitary or intratumoral dosing can maximize exposure at a resection bed, biopsy tract, or nodular residual lesion and may shorten the distance to cavity-adjacent tumor cells, but distribution may be less uniform and most concentrated along the cavity wall, catheter tract, or surgically accessible tumor interface [6, 17, 19]. At present, pediatric clinical data do not directly establish whether intraventricular versus intracavitary delivery produces superior deep tumor infiltration. For example, BrainChild-02 assigned resection-cavity versus ventricular delivery according to tumor anatomy and dissemination rather than as a randomized route comparison, making route-specific efficacy and infiltration conclusions hypothesis-generating [6]. Accordingly, future studies should stratify route by tumor location and dissemination pattern and should include route-specific correlatives to determine whether ICV, intrathecal, intracavitary, intratumoral, or combined dosing best supports cellular penetration into the intended tumor compartment [6, 17, 32]. 

### Toxicity, persistence, and management in the developing CNS

Safety is paramount in children whose brains are still maturing. Locoregional CAR‑T dosing has repeatedly shown favorable tolerability profiles with mostly grade 1–2 headaches, nausea, and transient focal symptoms whereasdose‑limiting toxicities have been rare in early pediatric studies of EGFR806 and B7‑H3 CARs delivered intracranially [6, 17]. When CRS does occur with systemic dosing, it appears to be dose‑dependent and manageable with algorithms adapted from hematologic settings [8]. Biological strategies can further mitigate risk while potentially improving efficacy and persistence: (*i*) using virus‑specific T‑cell backbones to provide physiologic costimulation prolongs persistence without lymphodepletion,^5^ (*ii*) incorporating 4‑1BB signaling improves persistence compared with first‑generation CARs in preclinical brain tumor models,^20^ and (*iii*) embedding an iC9 suicide switch allows prompt pharmacologic ablation of CAR‑T cells within the CNS if needed [34]. Of note, both the B7‑H3 and EGFR806 trials documented CAR detection in CSF or peripheral blood in some patients, establishing the feasibility of correlative persistence measures with locoregional delivery [6, 17]. 

### Resistance biology and rational combinations

Antigen escape and T‑cell dysfunction remain the dominant failure modes. Intratumoral heterogeneity will likely influence cellular therapy efficacy differently across platforms and tumor types. Conventional αβ CAR-T and TCR-engineered T-cell approaches can be highly potent but are most vulnerable when the selected surface antigen or peptide-HLA target is subclonal, spatially restricted, or downregulated under immune pressure. This concern may be relatively less pronounced in H3K27M-altered DMG when targets such as GD2 show high and comparatively uniform expression, but it is likely more important in medulloblastoma, ependymoma, ATRT, and recurrent high-grade glioma, where antigen density and expression can vary by tumor type, molecular subgroup, and disease compartment [22, 31, 32, 34, 35]. Multi-antigen CAR constructs, pooled or sequential CAR products, and serial antigen reassessment are therefore particularly relevant for embryonal tumors and ependymoma [13, 31, 32, 34]. In contrast, NK-cell and γδ T-cell platforms may partly compensate for antigen heterogeneity because they can integrate engineered CAR recognition with native innate-like tumor-sensing mechanisms, including EphA2/phosphoantigen-butyrophilin recognition by γδ T cells and GD2 CAR-NK activity in DIPG models [10, 36]. However, these less MHC-restricted mechanisms require careful CNS-specific safety assessment, because broader recognition does not by itself guarantee selectivity in the developing brain [10, 25, 37]. Thus, platform selection should be matched to disease biology: antigen-dense DMG may be well suited to GD2- or B7-H3-directed CAR programs, whereas spatially and molecularly heterogeneous medulloblastoma and ependymoma may benefit from multi-antigen CAR-T designs or complementary innate-like γδ/NK approaches [8, 10, 16, 20, 31, 32, 34, 36]. 

Target heterogeneity can be addressed by multiplexing (e.g., tandem/trivalent CARs to HER2/IL13Rα2/EphA2) or by sequencing complementary platforms (e.g., CAR‑T followed by TCR‑T targeting immunopeptidome‑defined neoepitopes), strategies supported by pediatric surfaceome/immunopeptidome maps.^13,24^ Exhaustion and poor metabolic fitness are the other cornerstone problems. A notable pediatric DMG advance is the demonstration that the dual IGF1R/insulin receptor inhibitor linsitinib reduces CAR‑T activation/exhaustion markers, increases central memory phenotype, and synergizes with GD2‑CAR T cells across in vitro and orthotopic models, which is an immediately testable combination given the clinical familiarity with IGF‑axis inhibitors [30]. These approaches align with emerging armoring and BBB‑focused tactics for brain tumor CAR‑T.^37^ Similarly, epigenetic modulation with tazemetostat can upregulate GD2 biosynthesis and sensitize medulloblastoma to GD2‑CAR killing, while preserving a CNS‑deployable safety switch through iC9 [34]. Looking ahead, antigen density tuning (radiation, epigenetic drugs), checkpoint blockade timed to avoid overlapping neuroinflammation, and metabolic reprogramming of CAR‑T products are converging avenues that should be explored in pediatric schedules tailored to minimize long‑term neurocognitive risk [25, 33]. 

### Disease‑specific implications

For DMG/DIPG, the weight of evidence now supports prioritizing GD2 and B7‑H3 programs delivered intracranially, with conservative intravenous dosing where systemic induction is used. The clinical signals (objective imaging responses and function gains), together with correlative CSF immunology suggest that repeated intraventricular dosing can generate a sustained, localized immune response in a space traditionally considered “immune‑cold”.^8,14,18^ For medulloblastoma, the field should deliberately test HER2‑CAR strategies leveraging intracranial delivery and second‑generation costimulation, while piloting GD2‑directed approaches in high‑risk SHH and Group 3/4 subtypes, including antigen‑upregulation tactics. Encouragingly, both αβ CAR‑T and γδ T approaches have preclinical rationales that are compatible with normal neural tissue safety [10, 13, 18, 19, 34]. Ependymoma presents a different operational challenge: while B7‑H3 is a strong target, durable responses may depend on pre‑emptive treatment at lower tumor burden, multimodal immune engagement, or novel binders (e.g., mAb806‑style cryptic epitopes) to overcome heterogeneous antigen presentation; preclinical‑to‑clinical translation should incorporate adaptive designs that allow early escalation to combination therapy in nonresponders [6, 31]. 

### Trial design, endpoints, and correlative science

The maturing pediatric experience provides actionable operational guidance [35, 38]. First, neurosurgical infrastructure enabling reliable, repetitive intracranial access is essential; details such as programmable shunt “virtual off” settings and standardized CSF handling reduce risk and unlock frequent dosing schemas [7]. Second, studies should embed prospective CSF proteomic/cytokine profiling and CAR tracking, which have already yielded mechanistic insights (e.g., CSF cytokine surges and CAR persistence associated with clinical benefit in DIPG) [7, 17]. Third, imaging criteria must accommodate therapy‑induced edema and pseudoprogression; pediatric trials have already encountered cases where peritumoral edema complicates response adjudication after intracranial CAR infusion [6]. Finally, where feasible, early integration of biologically guided combinations (e.g., linsitinib with GD2 CAR in DMG, tazemetostat with GD2 CAR in medulloblastoma) should be protocolized with predefined stopping and de‑escalation rules to preserve safety [30, 34]. 

Outside of the formal evidence-synthesis window, current clinical trial registrations illustrate how quickly the pediatric CNS cellular therapy landscape is diversifying. Registered studies include locoregional B7-H3 CAR T cells for pediatric primary CNS tumors (Loc3CAR; NCT05835687) [39], iC9-GD2 CAR T cells for relapsed/refractory pediatric CNS tumors (NCT05298995) [40], a quad-target locoregional product directed against B7-H3, EGFR806, HER2, and IL13-zetakine (BrainChild-04; NCT05768880) [41], an IL-7Rα–armored B7-H3 CAR T study for DIPG (NCT06221553) [42], intracerebroventricular GPC2-CAR T cells for medulloblastoma and other CNS embryonal tumors (NCT07087002) [43], and pediatric IL13Rα2 CAR T cells administered after lymphodepleting chemotherapy (NCT04510051) [44] Although these registry entries are not part of the extracted evidence set, they reinforce key near-term directions for the field: broader antigen coverage, repeated CNS-directed dosing, safety engineering, and biologically rational augmentation of CAR-T activity.

Beyond catheter-based locoregional administration, future strategies to improve CNS delivery may include transient BBB modulation with MR-guided focused ultrasound (MRgFUS). Preclinical work has shown that focused ultrasound can enhance delivery of targeted NK-92 cells to brain tumors and improve survival, and orthotopic DMG models have demonstrated increased therapeutic delivery and efficacy after BBB opening [45, 46]. Early pediatric DMG experience has also established the feasibility of neuronavigation-guided focused ultrasound BBB opening and drug delivery without sedation [47]. Although MRgFUS has not yet been established as part of pediatric adoptive cellular therapy protocols, it represents a rational adjunct for prospective study to increase regional trafficking, reduce the systemic dose required for activity, and potentially widen the therapeutic window.

### Limitations

Most clinical data remain early‑phase, single‑center, and small‑N, and many signals arise in multiple pretreated, late‑line patients with bulky disease, settings biased against cellular therapy. A further limitation is potential selection bias introduced by the logistics of early cellular therapy trials. Many studies are single-center, procedurally intensive, and slow to initiate because they require referral to a specialized center, eligibility confirmation, apheresis or cell procurement, product manufacturing and release testing, neurosurgical catheter placement, repeated intracranial dosing, and close protocolized monitoring. These requirements may preferentially select patients and families with greater socioeconomic resources, flexible employment, access to travel and lodging, English-language or health-system navigation advantages, and sufficient clinical stability to wait through manufacturing and travel. Conversely, patients with rapidly progressive disease, limited financial flexibility, long travel distance, caregiving constraints, or inability to remain near a study site may be underrepresented. This may limit generalizability, particularly for aggressive pediatric CNS tumors in which tempo of disease and treatment access are tightly linked. Larger multi-site trials, harmonized manufacturing logistics, remote pre-screening, travel and lodging support, and decentralization of selected monitoring visits may help mitigate this bias and improve external validity. Antigen prevalence estimates also vary with assay and tissue handling; single‑cell and proteogenomic confirmation should become standard. Preclinical models, often xenografts in NSG mice, do not replicate the human pediatric immune microenvironment. The syngeneic ependymoma models beginning to appear are a step in the right direction [31]. Nevertheless, the converging safety and feasibility data for intracranial dosing, together with the first durable responses in DMG, justify a cautious but decisive progression to multi‑site phase II studies with robust correlative science [6, 8, 17]. 

## Conclusion

Pediatric neuro‑oncology is witnessing a transition from feasibility to early efficacy for cellular therapies. The combination of pediatric‑specific antigen discovery, locoregional delivery, and mechanism‑based combinations is producing the first durable responses in diseases that were uniformly fatal. Near‑term priorities include (i) multi‑antigen CAR designs or platform sequencing to pre‑empt antigen escape; (ii) expansion of intracranial dosing into frontline consolidation for ultra‑high‑risk groups where tumor burden is lowest; (iii) prospective validation of pharmacologic “dimmer switches” (e.g., iC9) to maintain safety; and (iv) rigorous, child‑appropriate neurocognitive follow‑up to ensure that survival gains are not offset by long‑term harm. In parallel, emerging targets such as EphA3 and modality‑expanding platforms like γδ T cells should move swiftly but safely into pediatric trials, with correlative assays calibrated to pediatric immunobiology.

## Supplementary Information

Below is the link to the electronic supplementary material.

Supplementary Material 1 (DOCX 14.9 KB)

## Data Availability

This study did not use or generate primary research data. All extracted data from the included studies are presented in the tables within the manuscript and supplementary materials. Additional extracted information that was collected during the review process but not reported can be made available upon reasonable request by contacting the corresponding author.
