# Supplementary material for: Locoregional and systemic adoptive cellular therapies for pediatric brain tumors: a systematic review of CAR‑T, TCR‑engineered T cells, and NK cell strategies
Source: Neurosurg Rev. 2026 Aug 7;49(1):519. doi: 10.1007/s10143-026-04437-0 (PMC13451245; doi:10.1007/s10143-026-04437-0)
Supplement: Supplementary file 1 [file 10143_2026_4437_MOESM1_ESM.docx]

Supplemental table 1.

| **Database** | Full Search Syntax | Number of Search Results |
| --- | --- | --- |
| PubMed | ( ("chimeric antigen receptor*"[tiab] OR "CAR T"[tiab] OR "CAR-T"[tiab] OR "CAR T cell*"[tiab] OR "CAR-NK"[tiab] OR "CAR NK"[tiab] OR "natural killer cell*"[tiab] OR "NK-92"[tiab] OR "T-cell receptor*"[tiab] OR TCR[tiab] OR "TCR-engineered"[tiab] OR "adoptive cell*"[tiab] OR "adoptive cellular therap*"[tiab] OR "cell therap*"[tiab]) OR (Chimeric Antigen Receptor* [Mesh] OR Receptors, Antigen, T-Cell [Mesh] OR Immunotherapy, Adoptive [Mesh] OR Natural Killer Cells [Mesh]) ) AND ( ("brain tumor*"[tiab] OR "brain tumour*"[tiab] OR "brain neoplasm*"[tiab] OR glioma*[tiab] OR glioblastoma*[tiab] OR "high-grade glioma*"[tiab] OR "diffuse midline glioma*"[tiab] OR "diffuse intrinsic pontine glioma"[tiab] OR DIPG[tiab] OR DMG[tiab] OR medulloblastoma*[tiab] OR ependymoma*[tiab] OR "atypical teratoid rhabdoid tumor*"[tiab] OR ATRT[tiab]) OR (Brain Neoplasms[Mesh] OR Glioma[Mesh] OR Medulloblastoma[Mesh] OR Ependymoma[Mesh]) ) AND ( (pediatric*[tiab] OR paediatric*[tiab] OR child*[tiab] OR adolescen*[tiab] OR infant*[tiab] OR "young adult*"[tiab]) OR (Pediatrics[Mesh] OR Child[Mesh] OR Infant[Mesh] OR Adolescent[Mesh] OR Young Adult[Mesh]) ) AND ( HER2[tiab] OR ERBB2[tiab] OR "B7-H3"[tiab] OR CD276[tiab] OR EGFR[tiab] OR EGFRvIII[tiab] OR "EGFR 806"[tiab] OR EGFR806[tiab] OR GD2[tiab] OR "disialoganglioside"[tiab] OR "IL13RA2"[tiab] OR "IL13Rα2"[tiab] OR "interleukin 13 receptor alpha 2"[tiab] OR EPHA2[tiab] OR "EphA2"[tiab] OR CD70[tiab] ) | 52 |
| Embase | (("chimeric antigen receptor*":ti,ab OR "CAR T":ti,ab OR "CAR-T":ti,ab OR "CAR T cell*":ti,ab OR "CAR-NK":ti,ab OR "CAR NK":ti,ab OR "T-cell receptor*":ti,ab OR "TCR-engineered":ti,ab OR "TCR-T":ti,ab OR "adoptive cell therap*":ti,ab OR "adoptive cellular therap*":ti,ab OR "natural killer cell therap*":ti,ab OR "NK-92":ti,ab) AND ("pediatric brain tumor*":ti,ab OR "paediatric brain tumour*":ti,ab OR glioma*:ti,ab OR glioblastoma*:ti,ab OR "high-grade glioma*":ti,ab OR "diffuse midline glioma*":ti,ab OR "diffuse intrinsic pontine glioma":ti,ab OR DIPG:ti,ab OR DMG:ti,ab OR medulloblastoma*:ti,ab OR ependymoma*:ti,ab OR ATRT:ti,ab OR "atypical teratoid rhabdoid tumor*":ti,ab) AND (pediatric*:ti,ab OR paediatric*:ti,ab OR child*:ti,ab OR adolescen*:ti,ab OR infant*:ti,ab OR "young adult*":ti,ab) AND (HER2:ti,ab OR ERBB2:ti,ab OR "B7-H3":ti,ab OR CD276:ti,ab OR EGFR:ti,ab OR EGFRvIII:ti,ab OR "EGFR 806":ti,ab OR EGFR806:ti,ab OR GD2:ti,ab OR disialoganglioside:ti,ab OR IL13RA2:ti,ab OR "IL13Rα2":ti,ab OR "interleukin 13 receptor alpha 2":ti,ab OR EPHA2:ti,ab OR EphA2:ti,ab OR CD70:ti,ab)) AND [english]/lim | 196 |
| Scopus | ("chimeric antigen receptor*" OR "CAR T" OR "CAR-T" OR "CAR T cell*" OR "CAR-NK" OR "CAR NK" OR "natural killer cell*" OR "NK-92" OR "T-cell receptor*" OR TCR OR "TCR-engineered" OR "adoptive cell*" OR "adoptive cellular therap*") AND ("brain W/3 (tumor* OR tumour* OR neoplasm*)" OR glioma* OR glioblastoma* OR "high-grade glioma*" OR "diffuse midline glioma*" OR "diffuse intrinsic pontine glioma" OR DIPG OR DMG OR medulloblastoma* OR ependymoma* OR ATRT OR "atypical teratoid rhabdoid tumor*") AND (pediatric* OR paediatric* OR child* OR adolescen* OR infant* OR "young adult*") AND (HER2 OR ERBB2 OR "B7-H3" OR CD276 OR EGFR OR EGFRvIII OR "EGFR 806" OR EGFR806 OR GD2 OR disialoganglioside OR IL13RA2 OR "IL13Rα2" OR "interleukin 13 receptor alpha 2" OR EPHA2 OR EphA2 OR CD70) | 76 |
